# Probing ultrafast C–Br bond fission in the UV photochemistry of bromoform with core-to-valence transient absorption spectroscopy

**DOI:** 10.1063/1.5113798

**Published:** 2019-10-11

**Authors:** Benjamin W. Toulson, Mario Borgwardt, Han Wang, Florian Lackner, Adam S. Chatterley, C. D. Pemmaraju, Daniel M. Neumark, Stephen R. Leone, David Prendergast, Oliver Gessner

**Affiliations:** 1 Chemical Sciences Division, Lawrence Berkeley National Laboratory, Berkeley, California 94720, USA; 2 Institute of Experimental Physics, Graz University of Technology, 8010 Graz, Austria; 3 Department of Chemistry, University of California, Berkeley, California 94720, USA; 4 Stanford Institute for Materials and Energy Sciences, SLAC National Accelerator Laboratory, Stanford, California 94025, USA; 5 Department of Physics, University of California, Berkeley, California 94720, USA; 6 Molecular Foundry, Lawrence Berkeley National Laboratory, Berkeley, California 94720, USA

## Abstract

UV pump–extreme UV (XUV) probe femtosecond transient absorption spectroscopy is used to study the 268 nm induced photodissociation dynamics of bromoform (CHBr_3_). Core-to-valence transitions at the Br(3*d*) absorption edge (∼70 eV) provide an atomic scale perspective of the reaction, sensitive to changes in the local valence electronic structure, with ultrafast time resolution. The XUV spectra track how the singly occupied molecular orbitals of transient electronic states develop throughout the C–Br bond fission, eventually forming radical Br and CHBr_2_ products. Complementary *ab initio* calculations of XUV spectral fingerprints are performed for transient atomic arrangements obtained from sampling excited-state molecular dynamics simulations. C–Br fission along an approximately $C_{S}$ symmetrical reaction pathway leads to a continuous change of electronic orbital characters and atomic arrangements. Two timescales dominate changes in the transient absorption spectra, reflecting the different characteristic motions of the light C and H atoms and the heavy Br atoms. Within the first 40 fs, distortion from $C_{3v}$ symmetry to form a quasiplanar CHBr_2_ by the displacement of the (light) CH moiety causes significant changes to the valence electronic structure. Displacement of the (heavy) Br atoms is delayed and requires up to ∼300 fs to form separate Br + CHBr_2_ products. We demonstrate that transitions between the valence-excited (initial) and valence + core-excited (final) state electronic configurations produced by XUV absorption are sensitive to the localization of valence orbitals during bond fission. The change in valence electron-core hole interaction provides a physical explanation for spectral shifts during the process of bond cleavage.

## I. INTRODUCTION

Photochemical reactions are the result of concerted electronic-nuclear motion induced by light-matter interaction. Gaining a fundamental understanding of these coupled dynamics by combining ultrafast spectroscopy with first principles calculations is the driving force behind a fast growing field of research.^1,2^ For example, the UV photochemistry of halogen-containing molecules involves ultrafast carbon-halogen bond cleavage, forming radical species that are short-lived and highly reactive. Here, direct detection of transient species during the photoinduced dissociation of bromoform using core-to-valence transitions is demonstrated, which provides a sensitive probe of the evolving molecular electronic structure and nuclear geometry, with ultrafast time resolution.

**FIG. 1.**
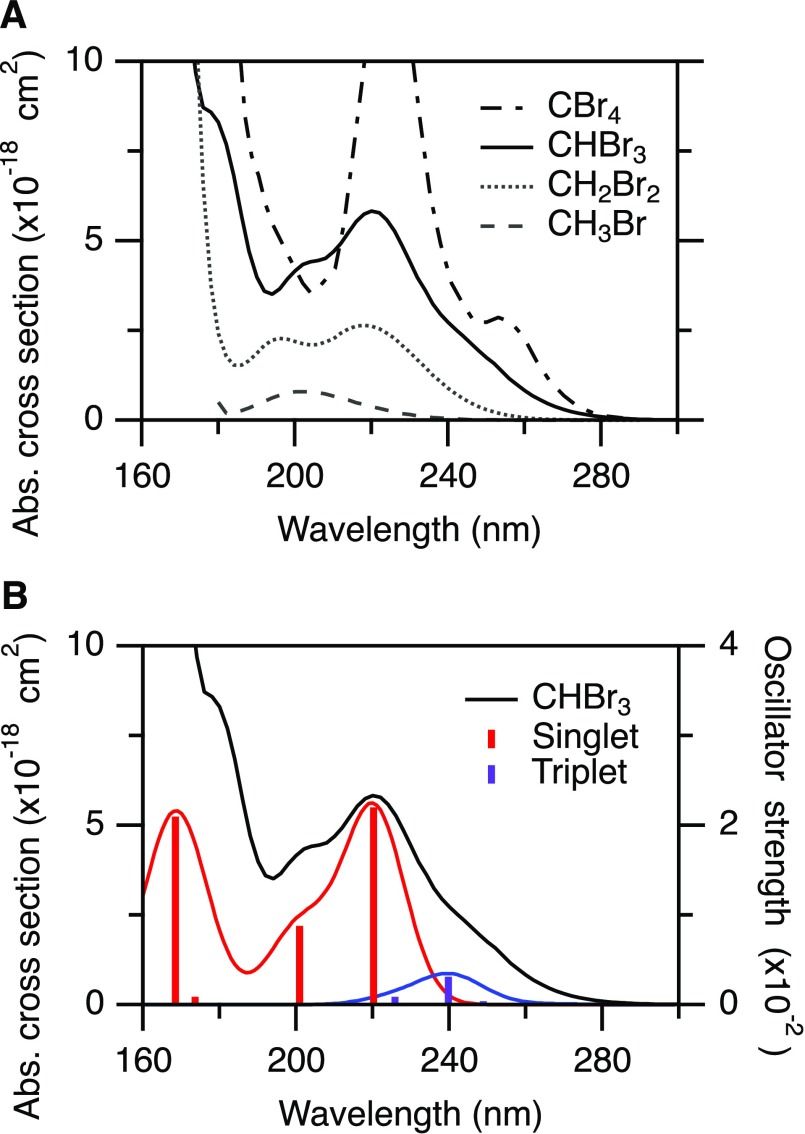
(a) Absorption spectra of bromomethanes: CH_3_Br (dashed), CH_2_Br_2_ (dotted), CHBr_3_ (solid), and CBr_4_ (dot-dash).^6^ (b) Absorption spectrum of CHBr_3_ (left, Gillotay *et al.*) and calculated oscillator strengths (right, Peterson and Francisco) for low-lying states of predominantly singlet and triplet character.^29,30^

The photochemistry of halogen-containing species has significant practical implications.^3^ For instance, bromoform is recognized as a key contributor to polar ozone depletion^4^ and is the largest source of bromine in the atmosphere due to a combination of increased absorption at longer wavelengths and considerable natural abundance.^5^ Increasing bromination of alkanes leads to an increase in absolute absorption cross section, new absorption bands, and a strong red-shift of the first absorption band as illustrated in Fig. 1(a), which shows gas-phase absorption spectra of CH_3_Br, CH_2_Br_2_, CHBr_3_, and CBr_4_.^6^ Absorption at longer wavelengths extends the photochemistry to lower altitudes.

**FIG. 2.**
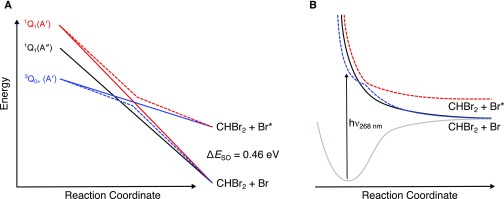
(a) Correlation diagram connecting the low-lying electronically excited states to photoproducts. Adiabatic (dashed) and diabatic (solid) correlations are shown. (b) Schematic adiabatic potential energy curves along the reaction coordinate.

Detailed knowledge of the UV photochemistry of haloalkanes, for example the CH_3_I photochemistry as summarized by Gardiner *et al.*,^7^ provides important building blocks for single-photon UV dissociation studies of gas-phase dihaloalkanes (CH_2_I_2_,^8–11^ CH_2_ICl,^12–15^ CH_2_BrCl,^16–18^ and CH_2_BrI^19–22^) and trihaloalkanes (CHBrCl_2_^23^ and CHI_2_Cl^24^). Generally, the photochemistry of the low-lying electronically excited states of haloalkanes arises from the promotion of an electron from a nonbonding pair of electrons on the halogen atom (X) to a σ^*^ antibonding orbital ($\sigma^{*}\leftarrow n$), resulting in rapid extension along the C–X bond coordinate.^25–28^ The absorption spectrum of bromoform is composed of diffuse overlapping features arising from multiple excited states [Fig. 1(b)]. *Ab initio* calculations by Peterson and Francisco place the first excited singlet state at λ < 234 nm, while the first triplet state is significantly lower in energy, accessible at λ < 297 nm.^29^ In Fig. 1(b), the calculated oscillator strengths are overlaid as stick spectra on the measured absorption spectrum of bromoform, as well as a broadened version of the same results after convolution with a 19 nm full width at half maximum (FWHM) Gaussian to facilitate comparison.^29,30^ As such, excitation at the red edge of the first absorption band of bromoform is likely dominated by excitation to states of predominantly triplet character, enabled by spin–orbit coupling with dipole-accessible singlet states.^29^ An adapted version of the correlation diagram by Merrill and co-workers for the UV photodissociation of CHBrCl_2_ is shown in Fig. 2(a), connecting the low-lying electronically excited states to photoproducts.^23^ Of particular importance is that only the ^3^Q_0_^+^(A′) and ^1^Q_1_(A′) surfaces (employing Mullikan's notation, $C_{s}$ symmetry labels in parentheses) lead to valence spin–orbit excited Br^*^ atom products. However, excitation to the ^1^Q_1_(A′) state (S_2_) lies higher in energy than ^3^Q_0_^+^(A′) and only becomes accessible at λ < 224 nm.^29^ The ^3^Q_0_^+^(A′) surface correlates diabatically with Br^*^ but adiabatically with Br due to an avoided crossing with the ^1^Q_1_(A′) surface. Note that the correlation diagram for polyhaloalkanes differs from that of monohaloalkanes as the reaction pathway has $C_{s}$ rather than $C_{3v}$ symmetry.^17^ Schematic adiabatic potential energy curves are shown in Fig. 2(b). Nonadiabatic dynamics allows a fraction of molecules dissociating on ^3^Q_0_^+^(A′) to undergo surface-hopping and form Br^*^ atoms.

Several product channels are energetically accessible following UV excitation^31^

$$\mathrm{CH}{\mathrm{Br}}_{3}+h\nu \to \mathrm{CH}{\mathrm{Br}}_{2}+\mathrm{Br}\;\lambda <488\;\mathrm{nm},$$ (I)

$$\to C{\mathrm{Br}}_{2}+\mathrm{HBr}\;\lambda <463\;\mathrm{nm},$$ (II)

$$\to \mathrm{CHBr}+{\mathrm{Br}}_{2}\;\lambda <338\;\mathrm{nm},$$ (III)

$$\to C{\mathrm{Br}}_{3}+H\;\lambda <309\;\mathrm{nm}.$$ (IV)

Broadly, two different viewpoints exist on the outcome of single-photon UV dissociation of bromoform within the first absorption band. Either only C–Br fission by channel (I) occurs, or both channel (I) and Br_2_ elimination by channel (III) are active. Br_2_ elimination was first suggested by Xu *et al.*, based on the detection of CHBr_2_^+^, CHBr^+^, and CBr^+^ products using vacuum ultraviolet (VUV) ionization time-of-flight mass spectrometry and imaging.^32^ Bayes *et al.* measured the Br atom yield between 266 and 344 nm by monitoring atomic fluorescence. While a unity quantum yield was observed at λ > 300 nm, the yield decreases to 0.76 ± 0.03 at 266 nm, which was interpreted as an indication that additional dissociation channels may be active at shorter wavelengths.^33^ Huang *et al.* detected Br_2_ via B–X band absorption following photolysis of bromoform at 248 nm using cavity ringdown spectroscopy.^34^ Contrasting these observations, multiple compelling pieces of evidence that only C–Br fission occurs upon 248 nm single-photon excitation have been presented by Zou *et al.*^35^ Using frequency modulated transient absorption to directly detect CHBr near 10841 cm^−1^ (1.344 eV), an upper limit for the CHBr yield of ∼0.003 was estimated. Second, the power dependence of Br_2_ products in photofragment translational spectroscopy experiments was shown to be quadratic (1.9 ± 0.1) with the photolysis laser intensity, consistent with a multiphoton origin of the Br_2_ fragments. Molecular elimination in polyhaloalkanes following multiphoton excitation is well documented.^36–38^

Upon photolysis, polyhaloalkanes can form stable isomers containing a carbon-halogen-halogen bond linkage, as was first demonstrated by Maier *et al.* employing cryogenic matrices.^39,40^ Formation of the isomer has been attributed to a recombination mechanism, where the environment confines the fragments and dissipates excess energy, eventually leading to the reassociation of the fragments into a local minimum isomer conformation.^41^ An alternative mechanism has been proposed, whereby ultrafast photoisomerization proceeds through a conical intersection that connects the lowest-lying singlet excited state of the parent molecule to the ground electronic state, forming the isomer en-route to dissociation.^42^ Subsequent ultraviolet-visible (UV-vis) transient absorption studies of gas-phase CHBr_3_ following 250 nm photolysis detected an absorption feature that forms and decays on timescales of 50 fs and 85 fs, respectively.^43^ It was hypothesized that the intermediate absorption corresponds to isomer formation.^43^ The proposed isomerization mechanism challenges the conventional understanding of haloalkane photochemistry, where a one-dimensional model of C–X bond extension is sufficient to capture the essential details of the dissociation rather than the full 3*N*-6 degrees of freedom that are, in principle, involved.^7^

Here, a UV pump–extreme UV (XUV) probe femtosecond transient absorption spectroscopy study of the 268 nm induced dissociation of CHBr_3_ is presented. Experimental spectra are complemented by high-level *ab initio* calculations of XUV spectral fingerprints of transient molecular species obtained from excited-state molecular dynamics (MD) simulations. The XUV spectra track the formation of singly occupied molecular orbitals (SOMO) in the photoexcitation step and the evolution of transient electronic configurations throughout the C–Br bond fission, eventually leading to the formation of radical Br and CHBr_2_ products. A global fit of the experimental XUV spectra indicates the formation and decay of an absorption feature on timescales of 40 ± 20 fs and 85 ± 10 fs, respectively. Comparison to first principles calculations reveals that the changes in the transient absorption spectra reflect the different characteristic timescales for motion of the light C and H atoms and the heavy Br atoms. Within the first 40 fs, distortion from $C_{3v}$ symmetry to form a quasiplanar CHBr_2_ by the displacement of the (light) CH alkane moiety causes significant changes to the excited state valence electronic structure. Displacement of the (heavy) Br atoms is delayed and requires up to ∼300 fs to form separate Br + CHBr_2_ products. A detailed discussion is provided on the physical origins of the observed shifts in inner-shell absorption energies.

## II. EXPERIMENTAL AND THEORETICAL METHODS

### A. Femtosecond XUV transient absorption spectroscopy

Experiments are performed using a femtosecond XUV transient absorption setup that has been described in detail previously.^44^ High-harmonic generation (HHG) produces a quasicontinuum of XUV by focusing 4.0 mJ, 35 fs, 1 kHz, 804 nm pulses with a 500 mm focal length lens into a semi-infinite gas cell containing 300 Torr of neon. The gas load is removed from the HHG propagation path using a differential pumping stage between the exit of the HHG cell and a layer of adhesive aluminum tape mounted 20 mm further downstream. The fundamental near-infrared (NIR) beam drills submillimeter diameter holes into the exit of the HHG cell and the aluminum tape. The volume between the two pinhole apertures is evacuated by a dry scroll vacuum pump. The XUV and NIR beams then enter a turbomolecular-pumped chamber where the NIR is rejected by a 200 nm thick aluminum foil. The transmitted XUV light impinges on a toroidal mirror that focuses the beam into an ∼4 mm long sample cell mounted inside a turbomolecular-pumped chamber. The sample cell consists of a Polytetrafluoroethylene (PTFE) capillary with ∼400 *μ*m apertures on opposing faces through which both the UV pump beam and the XUV probe beam enter and exit the cell.^45^ The sample cell is mounted on an XYZ translation stage and is resistively heated to ∼80 °C. Bromoform (Sigma Aldrich, 99%) is gently heated to 40 °C to increase the vapor pressure and delivered to the sample cell using stainless steel tubing. The vacuum chamber that houses the sample cell is isolated from the other vacuum chambers using 200 nm thick aluminum foils to prevent contamination of optics. The turbomolecular-pumped detector chamber houses an XUV spectrometer consisting of a variable line spaced grating (∼1200 lines/mm) and an X-ray CCD camera.

Static absorption spectra are recorded by measuring the XUV spectrum with the gas on/off. Transient absorption spectra are measured by introducing a pump pulse into the sample cell at an ∼2° angle relative to the XUV using a drilled-through mirror to transmit the XUV beam and reflect the pump beam. Downstream from the sample cell, a copper gasket with a small hole is used to transmit the XUV beam into the detector chamber and to dump the pump beam. In the pump arm, up to 4.0 mJ of NIR are used to generate UV pump pulses by frequency-tripling the fundamental. Second harmonic generation (SHG) is performed in a 0.1 mm thick, 29.2° β-barium borate (BBO) crystal to produce 402 nm pulses. The 804 nm and 402 nm beams are separated using a dichroic mirror. A half-wave plate rotates the NIR polarization to vertical for type-I mixing; a second dichroic mirror recombines the 804 nm and 402 nm beams collinearly in a 0.1 mm thick 44.3° BBO to produce up to 300 *μ*J pulses of UV light at 268 nm. The polarization of both the UV and the HHG beams is horizontal in the laboratory frame. The UV pulse energy is controlled using a combination of a half waveplate and a thin film polarizer to attenuate the fundamental NIR. No UV pulse compression is applied. Four high-reflective UV mirrors remove residual fundamental and 402 nm light prior to focusing the 268 nm beam into the sample cell with a 500 mm focal length lens. The UV beam is focused to a spot diameter of ∼100 *μ*m (FWHM). An optical chopper that intercepts the pump beam with a 50% duty cycle acts as the master clock for the experiment. The rising and falling edges trigger CCD exposures at 8 Hz to alternately record pump-on and pump-off spectra. The time delay between each pump and probe pulse is varied between −500 fs and +10 000 fs using a computer-controlled delay stage, with positive Δ*t* defined as the pump pulse preceding the probe pulse.

The UV-induced change in absorbance is calculated for each time delay Δt as

$$\Delta A(E,\Delta t)=-\log_{10}\frac{{I(E,\Delta t)}_{\mathrm{signal}}}{{I(E,\Delta t)}_{\mathrm{reference}}},$$ (1)

where ${I(E,\;\Delta t)}_{\mathrm{signal}}$ is the XUV spectrum recorded in the presence of the pump beam, while ${I(E,\;\Delta t)}_{\mathrm{reference}}$ is recorded with the pump beam blocked.

Spectral and temporal calibration of the setup is performed by filling the sample cell with xenon gas at pressures of ≪1 bar. The instrument response function (IRF) is determined by recording transient absorption spectra of xenon, whereby the intense UV pump pulse causes a ponderomotive shift of the absorption lines associated with *np* ← *4d* inner-valence to Rydberg excitations. A Gaussian fit to the time-dependent $6p\leftarrow {4d}_{5/2}$ absorption line shift is used to determine the zero delay point *t*_0_ and the FWHM of the instrument response function (IRF) of 110 fs. The XUV pulse duration is on the order of the NIR driving pulse duration or less. The UV pulse duration is significantly longer than the XUV pulse duration.

### B. *Ab initio* calculations

Excited-state MD simulations are performed by iteratively calculating the potential energy surface (PES) gradient using time-dependent density functional theory (TDDFT) and propagating the nuclei to follow the dissociation. XUV absorption spectra are calculated for a subset of nuclear configurations resulting from the MD simulations. The theoretical methodology follows the approach outlined for 1,3-cyclohexadiene.^2^ Note that in contrast to minimum energy pathways, the trajectories used here consider the momenta of the nuclei.

The initial conditions are obtained from a Boltzmann sampling of the Born-Oppenheimer molecular dynamics (BOMD) of the ground state molecule with a 300 K Nosé–Hoover thermostat as implemented in Q-Chem.^46,47^ The time step of the BOMD is 40 a.u. (∼0.96 fs) and the thermostat characteristic response time is 12 fs. Snapshots separated from one another by at least 48 fs are sampled as the starting coordinates and velocities to perform the excited-state MD with the fewest-switches surface-hopping (FSSH) algorithm.^48^ TDDFT surface-hopping dynamics are initiated on the lowest energy triplet (T_1_) excited-state PES. The Velocity-Verlet algorithm^49^ with a time step of 8 a.u. (∼0.19 fs) is used to evolve the nuclear coordinates, and the derivative-couplings between the lowest 8 triplet states are calculated to determine surface-hopping probabilities at each time step.

The trajectories are sampled every 40 a.u. (∼0.96 fs) to obtain the nuclear coordinates and the active PESs to calculate X-ray absorption spectra (XAS). Calculation of near-edge X-ray absorption fine structure (NEXAFS) spectra as implemented in NWChem^50^ involves a two-step approach. First, a reference valence excited state is modeled using the maximum overlap method (MOM);^51,52^ the resultant DFT eigenvectors and eigenvalues are then used in a restricted energy window (REW)^53^ linear-response TDDFT^54^ calculation to obtain the corresponding absorption spectrum. All calculations are carried out using the *def*2-tzvpd^55^ basis-set and the PBE0^56^ hybrid-DFT functional.

## III. RESULTS

**FIG. 3.**
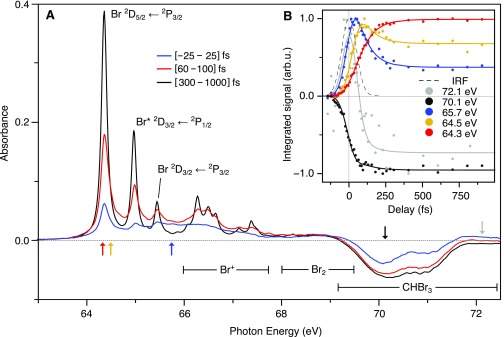
(a) UV pump–XUV probe transient absorption spectra at the bromine 3d edge of bromoform. Negative-going signal near 70 eV corresponds to depletion of the parent molecule. At early time [−25 to 25] fs (blue), a broad short-lived species near 66 eV is observed, at intermediate delays [60–100] fs (red) pronounced asymmetry in the neutral Br atom lines (64–66 eV) is observed, while after [300–1000] fs (black) the asymptotic CHBr_2_ + Br products are formed. Color-coded arrows indicate the energies selected to illustrate time-dependent spectral trends in panel B. (b) Circular points are measured data using the same color codes (energy specified in legend). The solid lines correspond to the result of a global fit analysis. The instrument response function (IRF) measured in xenon is overlaid (dashed gray).

The transient absorption spectra shown in Fig. 3(a) are obtained by integrating the measured ΔA(E,  Δt) curves according to Eq. (1) over separate pump-probe time-delay ranges as indicated in the figure legend. The pump pulse intensity in these measurements is 5 TW/cm^2^. Multiple spectral features and dynamic changes are readily apparent. Sharp absorption features at long delays correspond to neutral Br atoms that are probed by 3*d*^9^ 4*s*^2^ 4*p*^6^ ← 3*d*^10^ 4*s*^2^ 4*p*^5^ core-to-valence excitations. Both the core and valence orbital energies are split by spin–orbit coupling. Ground state Br atoms are probed by ^2^D_5/2_ ← ^2^P_3/2_ and ^2^D_3/2_ ← ^2^P_3/2_ transitions into spin–orbit-split core-excited states at 64.36 eV and 65.45 eV, respectively, while (valence) spin–orbit excited Br^*^ atoms are observed by the ^2^D_3/2_ ← ^2^P_1/2_ transition at 64.97 eV. Multiple sharp features in the range 66.0–67.5 eV correspond to absorption by Br^+^ ions, indicating contributions from multiphoton processes.^57^ At a lower pump intensity of ∼1 TW/cm^2^, the Br^+^ signal can be suppressed while the neutral Br signal remains, albeit with significantly reduced signal-to-noise. The absorbance scales approximately linearly with pump intensity for neutral Br atoms and greater than the square for Br^+^ ions, as indicated by Fig. S1 of the supplementary material. A negative change in absorbance beyond ∼69 eV arises from depletion of the parent molecule concentration by UV pump excitation. It appears as a broad bimodal absorption feature arising from Br core ${3d}_{5/2}$ and ${3d}_{3/2}$ to LUMO transitions in the parent molecule centered at 70.07 eV and 71.05 eV, respectively. No transient absorption features are observed between 68.3 eV and 69.0 eV up to the maximum delay of 10 ps (not shown). The absence of any signal in this range confirms that no molecular Br_2_ is formed within the timeframe of the experiment, since it would result in a spin–orbit-split, bimodal absorption feature in this energy range.^58^ As no inner-shell absorption cross section of Br_2_ could be located in the literature, an upper limit for Br_2_ formation is estimated by comparison to the CHBr_3_ depletion, assuming that the per-atom cross section is equal and including a $\frac{3}{2}$ weighting to account for the reduced number of Br atoms in Br_2_. This approach suggests an upper limit of [Br_2_]/[CHBr_3_] ≤ 0.04. Transient absorption spectra in Fig. 3(a) for short pump-probe delays, Δ*t* < 25 fs (blue), show a very broad absorption feature centered at ∼66 eV with a FWHM of ∼4 eV. Notable depletion of CHBr_3_ and some Br atom formation within this timeframe is also observed. At intermediate delays, Δ*t* = 60–100 fs (red), the neutral Br atom signal has reached approximately half the asymptotic intensity and a pronounced asymmetry in the lineshapes is observed in the form of shoulders on the high-energy sides of the peaks. Br^+^ ions form faster than neutral Br atoms, already reaching their asymptotic yield after ∼100 fs.

The time-dependent behavior of the XUV transient absorption spectra is explored in more detail in Fig. 3(b) by inspecting key spectral regions using the same colors as marked by color-coded arrows in Fig. 3(a). The circular data points in Fig. 3(b) have been derived by integrating the time-dependent signals across 0.1–0.5 eV wide spectral regions and their respective maxima are normalized to unity. Solid curves are the result of a fit procedure described in the following section. Depletion of the parent CHBr_3_ population, as indicated by the Br core ${3d}_{5/2}$–valence transition near 70.1 eV and emergence of Br atom fragments as indicated by the peak at 64.2 eV are shown in black and red, respectively. Several transients with different temporal trends are illustrated by cuts at 72.1 eV (gray), 65.7 eV (blue), and the intermediate Br peak shoulder near 64.5 eV (yellow). The signature of a fast appearing population near 66 eV reaches its maximum for time delays less than 50 fs, while appearance of the Br peak shoulder at 64.5 eV is delayed, reaching a maximum after ∼100 fs. The asymptotic yield of Br atoms is only reached after ∼300 fs. Further time delay/C–Br extension does not influence the atomic Br peak intensity, position or line shape, indicating that changes to the valence electronic environment have ceased at this point. The signal at 72.1 eV at first corresponds to additional absorption (positive signal), but it overlaps spectrally with the signal depletion caused by CHBr_3_ photolysis, which dominates for delays >100 fs so the overall signal in this window becomes negative. The IRF is overlaid as a dashed curve.

## IV. ANALYSIS

### A. Global fit procedure

**FIG. 4.**
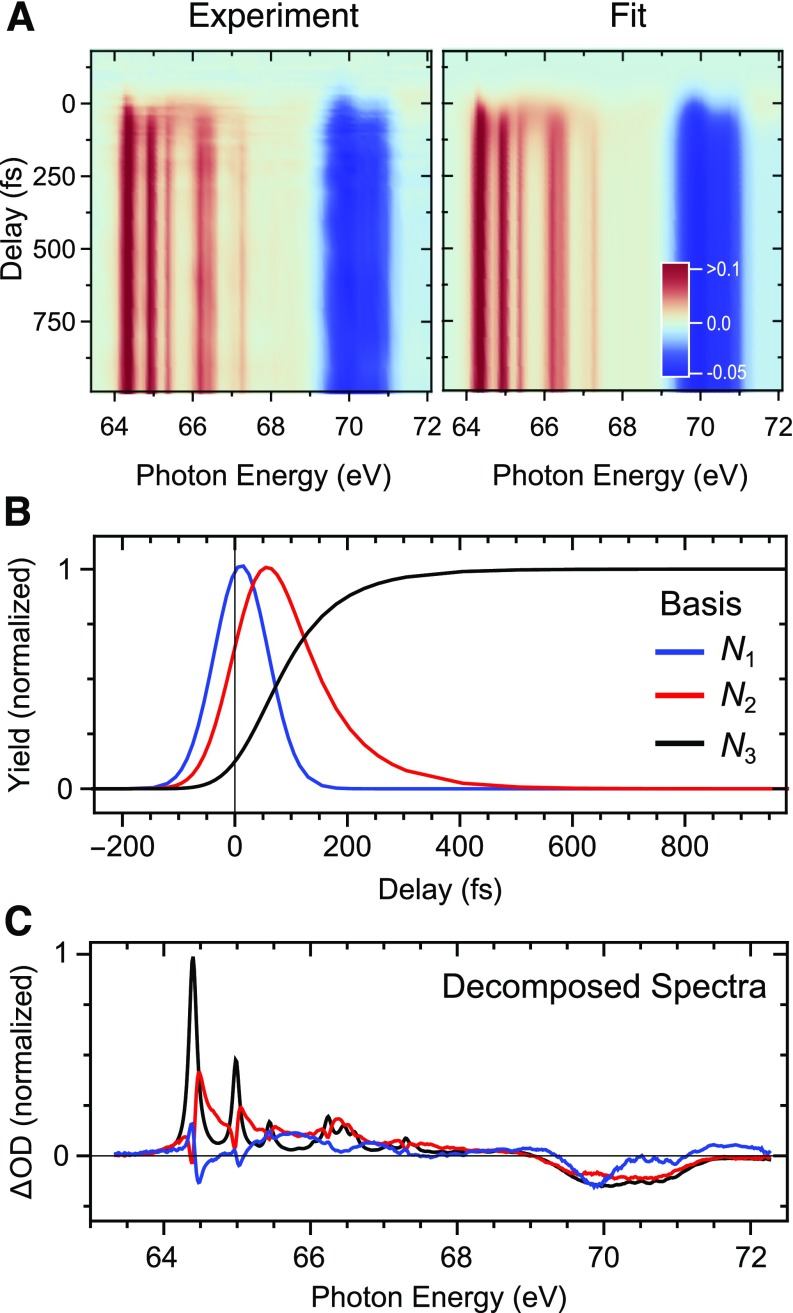
Global fit analysis of the time-resolved XUV absorption spectrum of CHBr_3_. (a) Measured (left) and global fit (right) data as a function of XUV absorption energy and pump-probe time delay. (b) The time basis set is defined by a three-state model where each state is sequentially populated, while the rate constants connecting the three states are free fit parameters. (c) Decomposed spectra.

In order to analyze the dynamic trends more quantitatively, the time-resolved spectra are decomposed into transient spectral contributions using a global fit procedure based on the standard least squares algorithm. The left panel in Fig. 4(a) shows a false-color representation of the measured ΔA(E,  Δt) curves as a function of energy (horizontal) and time (vertical) up to Δ*t* = 1000 fs for a pump pulse intensity of 5 TW/cm^2^. Signal depletion appears in dark blue, enhancement in orange, and red as indicated by the color bar. The acquired transient absorbance signal may be regarded as a data matrix (*T X A*) of dimensionality (t × e), where t denotes the number of sampled time delays and e the number of sampled energies. Under the working assumption that the shape of the absorption spectrum of each transiently populated state is time-independent and the population of each state is time-dependent, the data matrix can be decomposed into a spectrum matrix, *E*(n × e), and a time matrix, *Τ*(n × t), with n denoting the number of states, such that

$$\mathrm{TXA}=T^{T}E,$$ (2)

where the exponent *T* denotes the transpose of a matrix. This approach does not impose any constraints on the spectral shape related to a particular state and different kinetic models can be rapidly compared. A variety of different kinetic models have been evaluated to describe the data. A 1-step model clearly cannot accurately reproduce the observed dynamics. A 2-step model, however, is found to be sufficient to capture the dynamics (see the supplementary material for details). The heuristic approach to achieve the best description of the data with the least amount of free fit parameters leads to a time matrix composed of three states/components with time-dependent populations $N_{i}\left(i=1,\;2,\;3\right)$ that are sequentially populated and can be described by the following system of differential equations:

$$\frac{dN_{1}}{\mathrm{dt}}=g\left(t-t_{0},\;\sigma \right)-k_{1}N_{1}\left(t\right),$$ (3)

$$\frac{dN_{2}}{\mathrm{dt}}=k_{1}N_{1}\left(t\right)-k_{2}N_{2}\left(t\right),$$ (4)

$$\frac{dN_{3}}{\mathrm{dt}}=k_{2}N_{2}\left(t\right).$$ (5)

Here, $k_{i}$ ($\tau_{i}=1/k_{i}$) are the rates (timescales) for transitions between states *i* and *i *+* *1 and g(t) indicates a Gaussian-shaped initial population function accounting for the instrument response. The two rates $k_{1}$ and $k_{2}$ (and three time-independent amplitude factors) are free fit parameters, while g(t) is fixed to a Gaussian fit of the measured IRF. This approximation is feasible as the IRF is dominated by the pump pulse duration. Within this model, spectral component $N_{1}$ is initially populated by the pump pulse and decays into component $N_{2}$ with rate $k_{1}$, which decays to populate component $N_{3}$ with rate $k_{2}$.

The result of the fit procedure is shown in the right panel of Fig. 4(a). The time-dependent behavior of the fit and the experimental data are compared in detail in Fig. 3(b). The fit provides a very good description of the measured ΔA(E,  Δt) trends despite their rich structure and the limited number of free fit parameters. For example, the transients highlighted in Fig. 3(b) have different appearance times as well as different rise and decay dynamics but are all simultaneously captured by the relatively simple model. Using the sequential population model according to Eqs. (3)–(5), two timescales $\tau_{1,2}=\frac{1}{k_{1,2}}$ are necessary and sufficient to describe the data with $\tau_{1}=40\pm$ 20 fs and $\tau_{2}=85\pm 10$ fs. Excellent agreement is found with the time constants of 50 fs and 85 fs, respectively, observed in UV-visible transient absorption measurements at a substantially lower pump fluence of ∼0.04 TW/cm^2^, suggesting that the observed dynamics result from single-photon dissociation.^43^ Though we cannot definitively exclude contributions arising from multiphoton excitation, such as two-photon absorption to Rydberg states, we anticipate that their contribution to the atomic Br signal is minor. The merit of different kinetic models was compared using the statistical *F*-Test (see the supplementary material). The temporal basis set is shown in Fig. 4(b). The corresponding decomposed spectral components are shown in Fig. 4(c).

The spectra associated with the three components are largely overlapping but exhibit clear differences regarding the intensities, positions, and shapes of their absorption features. In the energy range corresponding to the bleach of the parent molecule, the components $N_{2}$ and $N_{3}$ are very similar whereas the initially populated component $N_{1}$ deviates. This finding indicates that in this spectral region, a decomposition based on only two components would already be sufficient. In contrast, in the photon energy range 64–68 eV, three spectral components are required. The red spectrum associated with component $N_{2}$ is reminiscent of the black spectrum related to the long-lived component $N_{3}$, but it is shifted to higher photon energies and broadened. The high-energy shoulders of the atomic peaks described above are entirely associated with the intermediate component $N_{2}$ while the final component $N_{3}$ spectrum exhibits virtually ideal Lorentzian lineshapes. Broad absorption features spanning almost the entire spectral range dominate the initially excited component $N_{1}$. Note that the sharp bipolar features in the range 64–65 eV coinciding with the main Br peaks are artifacts of the spectral decomposition due to significant overlap of signals in time and energy. Nevertheless, in the range between the atomic Br peaks, the decompositon yields the structure of the intermediate state.

### B. Spin-orbit branching

The detection of both Br and Br^*^ photoproducts following 268 nm excitation indicates that multiple potential energy surfaces participate in the dissociation of bromoform. Spin-orbit interaction can lead to the breakdown of the adiabatic approximation and coupling between otherwise orthogonal states. The dynamics for both Br and Br^*^ appear indistinguishable, possibly due to our 110 fs IRF. Two methods are used to independently determine the spin–orbit branching. The intensity ratio is first obtained by fitting the asymptotic Br and Br^*^ absorption peaks with Lorentzian functions, and then these are converted to amplitude by either (1) using the *ab initio* reduced transition dipole matrix elements given by Loh and Leone for the parallel polarization of pump and probe pulses used here,^59^ or (2) using the reported experimental absorption cross sections for the Br ^2^D_5/2_ ← ^2^P_3/2_, ^2^D_3/2_ ← ^2^P_3/2_ and Br^*^^2^D_3/2_ ← ^2^P_1/2_ transitions of 45.5 Mb, 5.4 Mb, and 54.3 Mb, respectively.^60^ For either method, a consistent Br/Br^*^ spin–orbit branching ratio of 3.7 ± 0.5 is found. Alternatively, the quantum yield of Br^*^ is found as Φ(Br^*^) = 0.21 ± 0.04, where

$$\Phi \left({\mathrm{Br}}^{*}\right)=\frac{\left[{\mathrm{Br}}^{*}\right]}{\left[\mathrm{Br}\right]+[{\mathrm{Br}}^{*}]},$$ (6)

for a pump intensity of 1 TW/cm^2^. A slightly larger value of Φ(Br^*^) = 0.23 ± 0.04 is obtained at 5 TW/cm^2^ but the two values agree within their uncertainties. A smaller spin–orbit branching ratio of 2.3 [or larger Φ(Br^*^) = 0.3] has been reported by comparison of translational energy distributions for Br and Br* to those of CHBr_2_ following nanosecond photolysis.^32^ However, the authors noted that the comparison for slow moving fragments was problematic.

### C. Theoretical calculations

*Ab initio* calculations by Peterson and Francisco suggest that excitation to the lowest-lying singlet state, Ã^1^A_2_, is unimportant at the red edge of the absorption band.^29^ First, the transition is dipole forbidden in $C_{3v}$ symmetry; consequently, the oscillator strength for this state is predicted to be negligible. Second, the vertical excitation energy is ∼5.1 eV (243 nm), which is higher in energy than the two lowest-lying triplet states Ã ^3^A_2_ and $\tilde{b}$ ^3^E at 4.6 and 5.0 eV (270 and 248 nm), respectively, transitions to which are dipole-allowed through spin–orbit coupling with close-lying singlet states.^29^ Accessing the second singlet state, $\tilde{B}$^1^E, from the ground state, which is a dipole-allowed transition, requires 5.6 eV (222 nm). Based on these calculations, we focus the discussion on dynamics that are initiated by populating the T_1_ electronic surface following absorption of a 268 nm photon.

**FIG. 5.**
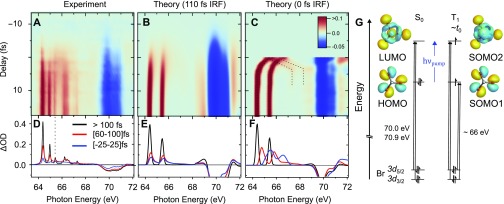
(a) Measured and (b) calculated TDDFT transient absorption as a function of XUV energy and pump-probe time delay. Negative-going signal (blue) near 70 eV corresponds to decreased absorption due to depletion of the parent molecule concentration by the pump pulse. Positive-going signal (red) corresponds to absorption features. The theoretical spectra have been broadened spectrally and temporally by convolution with Gaussian functions to match the experimental resolution. (c) Theoretical transient absorption without temporal broadening, dotted lines emphasizes the bifurcation of a spectral feature (see the text for details). Integration of (a)–(c) over pump-probe time delays specified in legend results in the spectra in panels (d)–(f), respectively. In (g), a schematic molecular orbital and XUV transition energy diagram is shown, tracing the evolution of the valence electronic occupation and structure.

Excited-state dynamics of bromoform are investigated by propagating trajectories on the T_1_ electronic surface using TDDFT with fewest-switches surface hopping (FSSH) and calculating the XUV absorption spectra for a subset of transient molecular configurations, as described in Sec. II A. Figure 5 compares the measured and calculated XUV absorption spectra, demonstrating remarkable agreement especially in capturing the dynamical evolution. Measured [Fig. 5(a)] and calculated [Fig. 5(b)] false-color maps show the spectral changes during the first few hundred femtoseconds using a similar color coding to that in Fig. 4. The TDDFT spectra are calibrated by a single energy offset (+0.5 eV) to agree with the experimental parent CHBr_3_ absorption peak position. Note that no spin–orbit splitting of the core Br(3*d*) manifold is included in the calculations. Instead, it is included after-the-fact by adding a replica of the computed spectrum that is shifted by the atomic spin–orbit splitting of ∼1 eV and rescaled by $\frac{2}{3}$ (according to ideal multiplicity differences in the spin–orbit split 3*d*_3/2_ and 3*d*_5/2_ channels). The production of valence spin–orbit excited Br^*^ atoms as well as multiphoton-induced Br^+^ ions is not included in the simulated XUV spectra. The calculated spectra have been convolved along the energy axis to account for lifetime broadening and the instrumental energy resolution with a Gaussian of 0.1 eV FWHM for transitions at <68 eV and 0.25 eV FWHM at >68 eV to match the Br atom and CHBr_3_ transitions. In Fig. 5(b), convolution with a Gaussian of 110 fs FWHM along the time axis has been used to match the temporal resolution of the experiment. For comparison, Fig. 5(c) shows the TDDFT spectra without temporal broadening. Integration over delay ranges of [−25 to 25] fs, [60–100] fs and [>100] fs leads to the extracted blue, red, and black spectra, respectively, for the measured [Fig. 5(d)] and calculated [Figs. 5(e) and 5(f)] results. Note the high quality of the calculated absorption energies, which is validated by the excellent agreement of the measured and calculated atomic Br line positions. The substantial impact of the IRF on the observed spectra and dynamics is highlighted by the comparison of Figs. 5(b) and 5(e) and 5(c) and 5(f). It demonstrates the importance of taking experimental boundary conditions into account when comparing theory and experiment. In Figs. 5(c) and 5(f) absorption near 66 eV at Δ*t* = 0 is seen to bifurcate with increasing time: the lower energy component increases in amplitude while the higher energy component declines and eventually disappears. The transient electronic configurations responsible for changes in inner-shell absorption during the photoinduced dissociation will be discussed below.

## V. DISCUSSION

XUV transient absorption at the Br(3*d*) edge probes the photoinduced chemistry from the viewpoint of the Br atoms since only transitions from well-localized Br(3*d*) inner-shell orbitals to valence orbitals exhibit notable oscillator strengths. A schematic molecular orbital and XUV transition energy diagram is shown in Fig. 5(g) for the ground state molecule and the photoexcited triplet state T_1_. Within this simplified picture, UV excitation transforms the originally fully occupied HOMO and unoccupied LUMO orbitals into singly occupied SOMO1 and SOMO2 orbitals. SOMO1 is derived from the Br(4*p*) orbitals that originally contribute to the HOMO in the parent molecule and asymptotically become atomic orbitals in the departing Br atom. Thus, XUV transitions into SOMO1 offer particularly detailed insight into the evolution of the electronic structure and the nuclear dynamics connecting the molecule to the isolated atom. SOMO2 develops localized character on the CHBr_2_ fragment providing a complementary viewpoint of changes in the molecular fragment. Throughout this work, shaded three-dimensional isosurfaces are rendered using an isovalue of 0.025 *e* where positive orbital values are colored in yellow and negative orbital values in green.

**FIG. 6.**
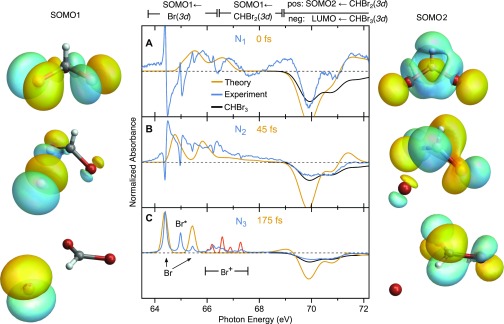
Decomposed spectra from the global fit of UV pump–XUV probe transient absorption spectra of bromoform recorded at the bromine 3d edge (blue) are compared to calculated TDDFT XUV absorption spectra (orange) for three different time delays. All spectra are normalized as described in the text to facilitate comparison. Calculated static absorption spectra of CHBr_3_ (black) and Br^+^ ions (red) are overlaid. (a) Decomposed experimental *N*_1_ and calculated 0 fs spectra highlight that short-lived species near 65.7 eV and >71 eV are observed corresponding to the SOMO1 ← 3d and SOMO2 ← 3d transitions, respectively. (b) *N*_2_/45 fs spectra illustrate the appearance of a pronounced high-energy shoulder on the neutral Br atomlike transitions for intermediate delays. (c) *N*_3_/175 fs spectra of the asymptotic CHBr_2_ + Br products. Isosurfaces are generated from a single trajectory in which slight distortion from $C_{3v}$ symmetry is visible at *t*_0_.

In the following, we provide a detailed discussion of the connections between changes in the transient absorption spectra and the orbitals involved in the transitions, providing deep insight into the coupled electronic-nuclear dynamics throughout the dissociation of CHBr_3_. Different stages of the dissociation are analyzed by comparing the $N_{1-3}$ decomposed spectra obtained from the global fit (blue) with calculated XUV absorption spectra (yellow) in Fig. 6. The blue and yellow spectra are normalized to the same local maxima in the 64–68 eV energy range (excluding the $N_{1}$ artifact at 64.4 eV) to facilitate comparison. The calculated spectra are convoluted along the energy axis as described in Fig. 5. No temporal broadening is applied to the calculated spectra. For reference, a vertically inverted copy of the static CHBr_3_ absorption spectrum is shown in black. The evolution of the SOMO1 and SOMO2 orbitals is shown alongside the spectra at each time snapshot. Note that increasing localization of SOMO1 onto the departing Br atom and SOMO2 onto the CHBr_2_ fragment occurs throughout the dissociation.

### A. Fast spectral evolution near *t*_0_

Transient spectra associated with very short delays are shown in Fig. 6(a). The fastest appearing spectrum, $N_{1}$, exhibits multiple absorption features. Neglecting the bipolar artifacts at energies <65 eV that arise from the spectrotemporal deconvolution procedure, two prominent features remain, a broad absorption near 66 eV and a more structured (positive and negative) absorbance at >69 eV. In a zero-order picture as illustrated in Fig. 5(g), the core–SOMO1 transitions are expected to be lower in energy compared to the parent molecule core–LUMO transitions by approximately the energy of the pump photon (4.63 eV). The broad ∼4 eV FWHM absorption band in $N_{1}$ near 66 eV is in agreement with this expectation. The overlaid calculated spectrum predicts a broad absorption spanning ∼65–67 eV. This broad absorption band comprises two types of transitions, one localized on the leaving atom, $\mathrm{CH}{\mathrm{Br}}_{2}\left({3d}^{20}\right)+\mathrm{Br}\left({3d}^{9}{4p}^{6}\right)\leftarrow \mathrm{CH}{\mathrm{Br}}_{2}\left({3d}^{20}\right)+\mathrm{Br}\left({3d}^{10}{4p}^{5}\right)$, and a second involving a Br(3*d*) orbital in the molecular fragment and the departing atom, $\mathrm{CH}{\mathrm{Br}}_{2}\left({3d}^{19}\right)+\mathrm{Br}\left({3d}^{10}{4p}^{6}\right)\leftarrow \mathrm{CH}{\mathrm{Br}}_{2}\left({3d}^{20}\right)+\mathrm{Br}\left({3d}^{10}{4p}^{5}\right)$. Note that these spectroscopic assignments are formulated in the separate atom limit to emphasize the distinction between the two contributions. During the dissociation, the bromine 4*p* orbitals predominantly form the molecular SOMO1 orbital. Each transition is split due to spin–orbit coupling of the inner-shell Br(3*d*_3/2_) and Br(3*d*_5/2_) orbitals, which are separated by ∼1 eV, to give a total of four Br(3*d*)–SOMO1 core-valence transition energies. At Δ*t* = 0, the two types of transitions for each spin–orbit component are approximately degenerate (exactly degenerate for $C_{3v}$ symmetry) and located near 65.5 eV and 66.5 eV for transitions from the Br(3*d*_5/2_) and Br(3*d*_3/2_) orbitals, respectively. The individual components overlap at early time in the average calculated XUV spectrum [Fig. 6(a)] due to the large number nuclear configurations sampled. The contribution of the different components to the total calculated XUV spectrum is illustrated in Fig. S5 for a single trajectory. For positive delays, the components quickly bifurcate upon increasing C–Br extension, with the atom-atom transition red-shifting and the molecule-atom transition blue-shifting and quickly fading [Fig. 4(c)]. As can be seen in Figs. 5 and 6, the calculations reproduce the rate at which the absorption features shift in energy very well. A quantitative comparison of the amplitudes at early and intermediate delays (i.e., for components $N_{1}$ and $N_{2}$) is partly hampered by the sharp artifacts from the spectral deconvolution. However, on a qualitative level, the relative amplitudes of atomic(like) and molecular transitions are also well captured, demonstrating the ability of the on-the-fly calculations to capture both the molecular dynamics as well as their XUV spectroscopic fingerprints.

The second absorption feature in the $N_{1}$ spectrum is observed at energies beyond ∼69 eV, overlapping with, and extending beyond, the core–LUMO transition energies of the parent molecule. It contains positive Δ*A*(*E*, Δ*t*) signals that partially overlap with the negative signals from the parent molecule depletion. The dynamics associated with the positive feature are highlighted by the gray curve in Fig. 3(b), which appears within the IRF, exhibits a maximum near Δ*t* = 0, and drops below zero for Δ*t* ≳ 100 fs. The global fit model confirms that positive absorption beyond ∼69 eV is only associated with the short-lived $N_{1}$ component [Fig. 6(a)], while the intermediate and final components $N_{2}$ and $N_{3}$ do not exhibit any positive signals in this spectral region [Figs. 6(b) and 6(c)]. A signal rise within the IRF may indicate that the component is associated with either purely electronic dynamics occurring prior to any nuclear motion (i.e., within the Franck-Condon region) or from dynamics that include only modest distortion relative to the parent molecular structure. The fast emergence of a signal in this range is consistent with the calculated spectra, which also predict the instantaneous appearance of an absorption feature associated with core–SOMO2 transitions in the photoexcited CHBr_3_ molecule.

### B. Spectral evolution at intermediate and long delays

At intermediate delays, corresponding to the $N_{2}$ spectral component in Fig. 6(b), the depletion signal beyond ∼69 eV resembles the inverted static CHBr_3_ spectrum shown in black. In the same energy window, the TDDFT calculations predict continued absorption from CHBr_2_-like species at >69 eV, overlapping with negative CHBr_3_ signals. The experimental data exhibit no substantial positive change in absorption in this region for intermediate and long delays. Note, however, that the absorption features in the experimental spectra in this region are substantially broader than the calculated spectra. Thus, the broad, intense negative signals may dominate over the smaller positive features. Additionally, transitions may occur at different energies and have different relative intensities than are estimated by theory.

The experimental spectra associated with long delays, corresponding to the $N_{3}$ spectral component in Fig. 6(c), differ from their intermediate $N_{2}$ counterparts only at energies <69 eV. In this region, features with a Lorentzian line shape emerge, consistent with atomic product formation. The quality of the calculated energies is validated by their agreement with the atomic Br line positions. However, the Δ*J* = 0 Br ^2^D_3/2_ ← ^2^P_3/2_ transition observed at 65.45 eV is significantly weaker than that predicted by the calculations. Additional spectral features observed in the range 65.9 eV–67.5 eV are reproduced in a separate simulation of multiphoton-induced Br^+^ ions [Fig. 6(c) red].

### C. Contributions to changes in transient XUV absorption spectra

**FIG. 7.**
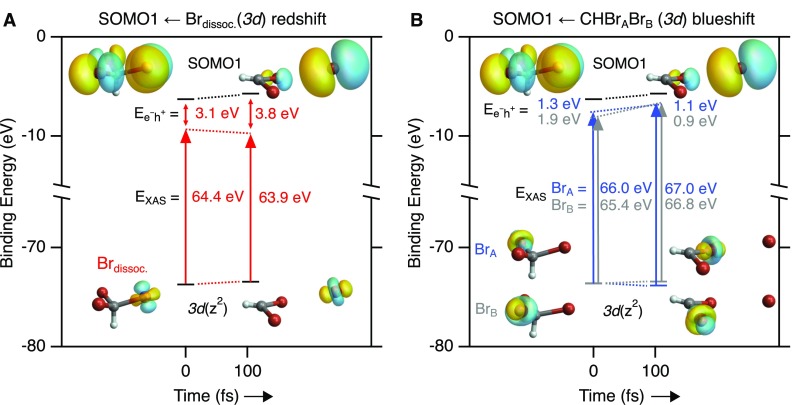
Calculated binding energies of the 3*d*_z_2 orbital for each Br atom and SOMO1 for two time steps: following T_1_ electronic excitation (Δ*t* = 0 fs) and after nuclear motion/dissociation (Δ*t* = 105 fs). Isosurfaces of the T_1_ orbital electron densities are shown. Note that in this particular trajectory, the molecule is slightly distorted from $C_{3v}$ symmetry at *t*_0_. SOMO1 becomes increasingly localized on Br_dissoc_ as the dissociation proceeds. Transition energies (E_XAS_) calculated using TDDFT are shown. XUV transitions result in a separation between the final state electron (e^−^) and hole (h^+^) pair. The change in electron-hole interaction ($E_{e^{-}h^{+}}$) is responsible for the redshift of transitions on the departing Br atom (a) and a blueshift of transitions from the Br atoms in CHBr_2_ to the increasingly localized valence orbital on the departing Br atom (b).

The spectra presented above illustrate that inner-shell transient absorption is sensitive to changes in both electronic structure and atomic configurations. The experimental signatures are amenable to comparison with high-level *ab initio* calculations, providing a rigorous approach to distinguish photochemical reaction mechanisms. Generally, inner-shell absorption energies correspond to the total energy differences between initial and final state electronic configurations. Predicting these differences is often challenging due to the breakdown of the single active electron picture, i.e., principally the response of all electronic orbitals in an atom or molecule needs to be taken into account. In many cases, however, it is instructive to discuss the transition energies in a simplified picture of core- and valence-orbital energies with the inclusion of an electron-hole interaction term, often referred to as exciton binding energy. This approximation is possible when the transition involves states that are dominated by a single orbital. This picture is illustrated in Fig. 7 for two types of transitions into the SOMO1 valence orbital, one from a 3*d* orbital on the Br atom that is departing the molecule [Fig. 7(a)] and the other for transitions from the 3*d* orbitals in the molecular CHBr_2_ fragment [Fig. 7(b)]. Note that in this particular example, the molecule is already slightly distorted from $C_{3v}$ symmetry at *t*_0_ to better illustrate the distinction between the atomic and molecular fragments at all times. In each panel, energy levels and isosurfaces of electronic orbitals are displayed for two showcase examples, i.e., the T_1_ excited parent molecule and near the free atom limit. Horizontal lines indicate the energies of the 3*d*_z_^2^ inner-shell orbitals and the SOMO1 valence orbitals. We choose to track the 3*d*_z_^2^ orbital as its isosurface is readily distinguished from other 3*d* orbitals throughout the dissociation. The core-to-valence energy difference in the valence-excited state, calculated transition energies (E_XAS_), and electron-hole binding energies in the XUV-excited final states ($E_{e-h^{+}}$) are summarized in Table I. Note that no rigid energy shift has been applied to E_XAS_ to match the experimental line positions in contrast to the spectra shown in Figs. 5 and 6 as this would require either the orbital or the electron-hole energy to be arbitrarily adjusted.

**TABLE I.** Valence-excited core 3*d*_z_^2^ energies, core 3*d*_z_^2^-SOMO1 difference energies, calculated transition energies (E_XAS_), and electron-hole pair interaction energies for transitions originating from different Br atoms (all energies in electron-volt). No rigid shift of E_XAS_ to match experimental transition energies is applied.

		Δ*t* = 0 fs	Δ*t* = 105 fs	Δ(*t*_105_ − *t*_0_)
	SOMO1	−6.28	−5.73	0.55
**Br_dissoc_**	**Core 3*d*_z_^2^**	−**73.73**	−**73.45**	**+0.28**
	ΔE_core-SOMO1_	67.45	67.72	+0.27
	**E_XAS_**	**64.38**	**63.93**	**−0.45**
	e-h^+^	3.07	3.79	+0.72
**Br_A_**	**Core 3*d*_z_^2^**	**−73.60**	**−73.83**	**−0.23**
	ΔE_core-SOMO1_	67.32	68.10	+0.78
	**E_XAS_**	**66.03**	**67.03**	**+1.00**
	e-h^+^	1.29	1.07	−0.22
**Br_B_**	**Core 3*d*_z_^2^**	**−73.54**	**−73.42**	**+0.12**
	ΔE_core-SOMO1_	67.26	67.69	+0.43
	**E_XAS_**	**65.38**	**66.78**	**+1.40**
	e-h^+^	1.88	0.91	−0.97

Within the picture illustrated in Fig. 7, the XUV transition energies correspond to the differences between core- and valence-orbital energies minus the electron-hole binding energies of the XUV-excited states. For example, 3*d*–SOMO1 transitions localized on the departing Br_dissoc_ atom redshift by ∼0.5 eV during the dissociation [Fig. 7(a)], while corresponding transitions involving Br_A_ and Br_B_ in the remaining molecular fragment blueshift by ≥1 eV [Fig. 7(b)], leading to the bifurcation of inner-shell absorption signals near ∼66 eV illustrated in Fig. 5(c). The different signs of the shifts originate from the change in electron-hole binding energy. We note that an alternative and equivalent description to changes in Br(3*d*) inner-shell absorption energies during dissociation has been presented recently,^61^ where the valence-excited (initial state) and (valence+)core-excited (final state) potentials of a molecule (HBr) along the interatomic coordinate are first calculated and subsequently the absorption strengths are determined. Changes to the electronic configuration of both the valence and core orbitals that result from inner-shell absorption are included in the core-excited potential. Here, however, we choose a different representation to illustrate the physical underpinnings of the transition energies and strengths associated with the localization of SOMO1 into Br_dissoc_(*4p*) as the dissociation proceeds. Note that both before and after dissociation the energy gap between the Br_dissoc_(3*d*)/CHBr_2_(3*d*) orbitals and SOMO1 in the valence-excited T_1_ state is consistently ∼67.5 eV (Table I), yet the transitions appear at distinct energies and show different trends owing to the different magnitude and sign of the electron-hole interaction term. Viewed from each Br atom, core-excitation produces a valence electron-3*d* hole pair whose spatial extent depends on the evolving valence orbitals. The sensitivity of the probe to ongoing changes to the valence chemical environment, in terms of absorption energy and strength, is governed by both the energy difference between, and overlap of, the initial and final states.

The results presented in Fig. 7 and Table I also demonstrate that different inner-shell spectroscopy techniques may exhibit different degrees of sensitivity with respect to a particular chemical transformation. For the specific case of UV-induced dissociation in bromoform, our calculations indicate that the Br(3*d*) core-level shifts of each distinct Br atom differ by only ∼0.1 to ∼0.3 eV across the dynamical range presented, whereas the XAS transition energies exhibit variations from a 0.45 eV redshift to a 1.4 eV blueshift. Intuitively, one might expect the opposite: that core-level shifts would be systematically larger than corresponding shifts in XAS peak positions, due to cancelation of shifts in the same direction for both the core-level and core-excited molecular orbital binding energies. Clearly, this is not the case here. Energetics, however, are not the only aspect to consider. While photoionization is always an allowed process at sufficiently high photon energies, XAS is subject to more restrictive selection rules and requires spatial overlap of the core- and valence-orbitals participating in a transition. At Δ*t* = 0, all localized 3*d* electron densities overlap with the delocalized SOMO1 orbital. At Δ*t* = 105 fs, however, only the Br_dissoc_ 3*d* orbitals are in the vicinity of the, now localized, SOMO1 while its overlap with the Br_A_/Br_B_ 3*d* orbitals vanishes, leading to rapid loss of the high-energy branch in the bifurcated Br(3*d*)–SOMO1 signal in Fig. 5(c).

### D. Trajectories and isomerization pathways

**FIG. 8.**
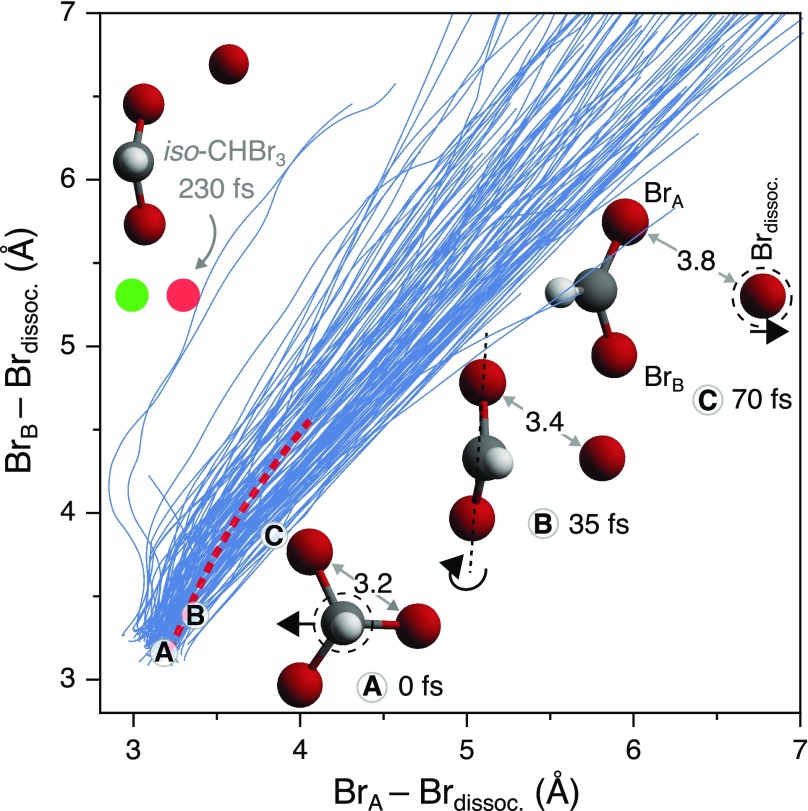
Correlation between the distance of the departing atom (Br_dissoc_) and each remaining Br atom of the CHBr_2_ fragment (labeled Br_A_/Br_B_). The diagonal represents a pathway with the least isomerization, where the Br_A_ and Br_B_ atoms remain equidistant to the leaving atom. The TDDFT trajectory calculations initiated on the T_1_ electronic surface (blue) show that dissociation is most-often equidistant, with a Gaussian distribution about the diagonal. 2 of 113 trajectories approach the vicinity of the minimum energy iso-CHBr_3_ geometry on the T_1_ surface (green circle) and the reported MS-CASPT2 iso-CHBr_3_ S_0_ geometry (red circle).^43^ The MS-CASPT2 trajectory (dashed red) falls within the distribution of symmetrical trajectories.^43^

Having established the ability of the *ab initio* XUV spectra to capture the temporal evolution of the experimental XUV spectra, we now analyze the underlying nuclear dynamics in greater detail by inspection of the MD trajectories from which the spectra are obtained. Figure 8 shows the distances between the departing atom (Br_dissoc_) and each remaining Br atom (Br_A_/Br_B_) in the CHBr_2_ fragment for 113 simulated TDDFT trajectories (blue traces) initiated on the T_1_ electronic surface. The diagonal represents a symmetrical pathway where the Br_A_ and Br_B_ atoms remain equidistant to the leaving Br_dissoc_ atom throughout the dissociation. As Br_A_ and Br_B_ are experimentally indistinguishable, trajectories are plotted such that they appear predominantly above the diagonal. The trajectories are available in the supplementary material. The pictographs in the lower half of Fig. 8 illustrate the atomic displacements during the early stages of a particular trajectory, starting with the initial $C_{3v}$ geometry of the parent molecule at 0 fs, marked (A). As a consequence of the high relative mass of the halogens, the initial motion is primarily associated with the lighter C and H atoms moving away from a tetragonal geometry toward a trigonal planar CHBr_2_ configuration within ∼35 fs (B). This momentum causes persistent rotation of the CHBr_2_ moiety around the Br_A_–Br_B_ axis. After 70 fs, near the maximum of the $N_{2}$ population in the global fit [Fig. 4(b)], the distances between the departing and each of the remaining Br atoms have extended by only ∼0.6 Å or ∼20% of the original distances. However, rotation around the Br_A_–Br_B_ axis has swept the plane of the CHBr_2_ away from the departing Br atom (C). The corresponding perturbation of the valence electronic structure is reflected in the XUV spectra by a spectrum that is characteristic of this dissociation-in-progress [Fig. 6(b)], illustrating the sensitivity of inner-shell spectroscopy to coupled electronic-nuclear dynamics. As the Br–CHBr_2_ distance increases, the purely atomic transitions gradually emerge [Fig. 6(c)].

The path taken by the MS-CASPT2 trajectory reported by Mereshchenko *et al.* is overlaid in Fig. 8 by a red dotted line; while being somewhat asymmetric, it falls within the distribution of direct C–Br fission TDDFT trajectories.^43^ Of the 113 TDDFT trajectories calculated here, two trajectories did approach the relaxed iso-CHBr_3_ geometry minimum shown as a green circle for the T_1_ surface. This T_1_ TDDFT isomer minimum geometry is similar to the S_0_ MS-CASPT2 minimum reported in Ref. 43, marked here as a red circle. The ensemble of TDDFT trajectories exhibit a Gaussian distribution about the diagonal, quantified in Fig. S6, i.e., symmetrical dissociation is the most probable outcome. While the exact branching will depend on the accuracy of the PES, isomerization appears to be strongly disfavored in the 268 nm photochemistry of isolated bromoform molecules.

**FIG. 9.**
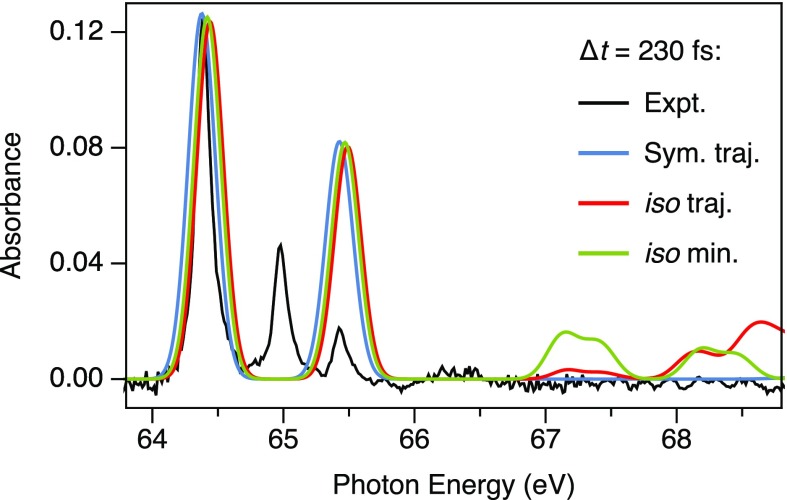
The experimental XUV absorption spectrum at a pump–probe delay of 230 fs (black) is compared to TDDFT XUV absorption spectra at the same time delay. The red spectrum corresponds to a trajectory that samples the formation of the iso-CHBr_3_ conformation (approximately red dot, Fig. 8), the blue spectrum to a typical trajectory following symmetric dissociation. The calculated XUV spectrum for the relaxed iso-CHBr_3_ T_1_ minimum geometry is also shown (green) (green dot, Fig. 8). The calculated iso-CHBr_3_ spectrum has additional absorption features near 67–68 eV arising from the molecular bonding character of the C–Br–Br linkage. The time evolution of the calculated spectra for symmetric dissociation is consistent with that observed in the experimental spectra.

The time scale for isomerization in the trajectory calculations, a rearrangement that requires significant motion of the heavy Br atoms, is much longer than the characteristic time constants on which the XUV spectra evolve, 40 ± 20 fs and 85 ± 10 fs, as obtained from a global fit. For example, the trajectory that passes through the iso-CHBr_3_ minimum conformation in Fig. 8, reaches this point after 230 fs. In order to test the prediction of the MD simulations that iso-forming trajectories are strongly suppressed, their expected spectroscopic fingerprints are compared with experimental observations. Figure 9 shows the experimental XUV absorption spectrum at a pump–probe delay of 230 fs (black) recorded at a relatively low pump intensity of 1 TW/cm^2^ to ensure that single-photon features dominate the spectrum. The measurement is compared to calculated XUV spectra for trajectories sampling either the iso-CHBr_3_ conformation (red, iso traj.) or following an approximately symmetric dissociation (blue, sym. traj.). XUV spectra for the relaxed iso-CHBr_3_ minimum conformation (green dot, Fig. 8) are also shown (green, iso min.). The XUV spectra obtained for the isomer minimum energy conformation are consistent with the spectra obtained from the isomer-sampling trajectory and only differ in the relative intensities of specific features. Note that the Br* and Br^+^ channels are not included in the trajectory calculations, and they are therefore absent in the calculated XUV absorption spectra. The calculated iso-CHBr_3_ spectra predict characteristic absorption features arising from 3*d*–SOMO1 transitions of the Br_2_-like C–Br–Br linkage at ∼68 ± 1 eV. No such feature is observed in the experiment. Instead, the measured spectrum is consistent with the XUV spectrum derived from a symmetrical dissociation pathway. The spectral fingerprints of specific molecular conformations, therefore, confirm that photodissociation of CHBr_3_ following the isomerization pathway is insignificant compared to the symmetric breakup channel.

Returning to the conflicting reports of Br_2_ as a primary photoproduct of bromoform, we have spectroscopically determined that no Br_2_ forms in a collision free environment on timescales <10 ps, i.e., no excited state directly dissociates to form Br_2_. The trajectory calculations are consistent with this observation, leading solely to C–Br fission, no other product channels were obtained. While it is possible that unimolecular dissociation on S_0_ could form Br_2_ over longer time scales, the molecular channel (III) is unfavorable as the radical channel (I) lies 1.14 eV lower in energy. Kalume *et al.* indicate that only 4% branching to molecular products at 248 nm is anticipated.^62^ Branching to form molecular products from unimolecular dissociation on S_0_ following photolysis at 268 nm would be even less favorable. To explain the reduced quantum yield of channel (I) as reported in Ref. 33, experiments probing radiative relaxation may prove valuable.

## VI. CONCLUSIONS

Unprecedented insight into the 268 nm-induced photodissociation of bromoform is reported. The element-specific nature of inner-shell spectroscopy is used to monitor the evolution of the valence electron environment of the different Br atoms from a well-localized perspective. A global fit model is used to decompose the XUV spectral evolution. Transformation of the initially populated excited state into photoproducts can be described by two sequential steps characterized by transition timescales of 40 ± 20 fs and 85 ± 10 fs. However, despite the ability of the 3-state model to accurately describe the experimental data, we caution that sampling continuous spectral changes with a finite experimental time-resolution may suggest the appearance of intermediate species but does not necessarily provide the most accurate description of the underlying physics. Here, the dynamics are investigated using excited-state MD simulations initiated on the T_1_ surface from which XUV absorption spectra are calculated. Analysis of the trajectories shows a dominant propensity for a continuous C–Br extension along the $C_{s}$ symmetry plane where the departing Br atom remains roughly equidistant to the other two Br atoms. The observed transient species correspond to the ongoing extension of the C–Br bond rather than a well-defined intermediate nuclear configuration. In particular, a previously proposed ultrafast “roaming” pathway that includes the transient formation of a BrCHBr–Br isomer is not reproduced. The absence of this pathway is further supported by the lack of any spectral fingerprint associated with the carbon-halogen-halogen arrangement at time delays where isomer formation is considered feasible based on the MD simulations. Dissociation through an isomer nuclear configuration is expected to occur rarely (only 2 out of 113 trajectories approached iso-CHBr_3_). Future ultrafast X-ray spectroscopy experiments with the intent of probing crucial early time photochemical signatures such as nonadiabatically coupled structural and electronic dynamics will require development of improved time resolution through ultrashort UV pulse generation.^63^

## SUPPLEMENTARY MATERIAL

See the supplementary material for a detailed description of the global fit models and residuals. TDDFT trajectories are also included.
